# A cross‐sectional study of avian influenza A virus in Myanmar live bird markets: Detection of a newly introduced H9N2?

**DOI:** 10.1111/irv.13111

**Published:** 2023-02-22

**Authors:** Laura K. Borkenhagen, Poe Poe Aung, Thura Htay, Zaw Win Thein, Ommar Swe Tin, Thet Su Mon, Win Myint, Emily S. Bailey, Timothy G. Wanninger, Ahmed M. Kandeil, Richard J. Webby, Gregory C. Gray

**Affiliations:** ^1^ Duke Global Health Institute Duke University Durham North Carolina USA; ^2^ Division of Infectious Diseases Duke University School of Medicine Durham North Carolina USA; ^3^ Duke Global Health Institute Myanmar Program Yangon Myanmar; ^4^ National Health Laboratory, Department of Medical Services Ministry of Health Yangon Myanmar; ^5^ Special Disease Control Unit Department of Public Health, Ministry of Health Naypyitaw Myanmar; ^6^ Livestock Breeding and Veterinary Department Ministry of Agriculture, Livestock and Irrigation Yangon Myanmar; ^7^ Department of Microbiology and Immunology University of Texas Medical Branch Galveston Texas USA; ^8^ Department of Infectious Disease St. Jude Children's Research Hospital Memphis Tennessee USA; ^9^ Center of Scientific Excellence for Influenza Viruses National Research Centre Giza Egypt; ^10^ Department of Medicine (Infectious Diseases) University of Texas Medical Branch Galveston Texas USA

**Keywords:** H9N2, influenza virus, live bird market, Myanmar, One Health

## Abstract

**Background:**

Zoonotic influenza surveillance in Myanmar is sparse, despite the risks of introduction of such viruses from neighboring countries that could impact the poultry industry and lead to spillover to humans.

**Methods:**

In July and August 2019, our multi‐institutional partnership conducted a One Health‐oriented, cross‐sectional surveillance (weekly for 3 weeks) for influenza A and influenza D viruses at the three largest live bird markets in Yangon, Myanmar.

**Results:**

The 27 bioaerosols, 90 bird cage swabs, 90 bird oropharyngeals, and 90 human nasopharyngeal samples yielded molecular influenza A detections in 8 bioaerosols (30.0%), 16 bird cages (17.8%), 15 bird oropharyngeals (16.7%), and 1 human nasopharyngeal (1.1%) samples. No influenza D was detected. Seven of the influenza A virus detections were found to be subtype A/H9N2, and one human nasopharyngeal sample was found to be subtype A/H1pdm. Among all IAV‐positive samples, three of the A/H9N2‐positive samples yielded live viruses from egg culture and their whole genome sequences revealing they belonged to the G9/Y280 lineage of A/H9N2 viruses. Phylogenetic analyses showed that these A/H9N2 sequences clustered separately from A/H9N2 viruses that were previously detected in Myanmar, supporting the notion that A/H9N2 viruses similar to those seen in wider Southeast Asia may have been introduced to Myanmar on multiple occasions.

**Conclusions:**

These findings call for increased surveillance efforts in Myanmar to monitor for the introduction of novel influenza viruses in poultry, as well as possible reassortment and zoonotic virus transmission.

## 1 BACKGROUND

Early warning screening and surveillance systems are essential to detect highly pathogenic and emerging zoonotic influenza strains at poultry markets. Despite being the largest mainland country in Southeast Asia, compared with neighboring countries, Myanmar has sparse surveillance for avian influenza A viruses (IAVs).^1^ Yet, multiple incursions of highly pathogenic avian influenza (HPAI) A/H5N1 strains have caused considerable poultry losses since they were first detected in 2006^2^, ^3^ and again in 2017–2018.^4^ Additionally, an incursion of A/H9N2 was detected at live bird markets in Yangon in 2015.^5^ In the year following these first detections of A/H9N2 in Myanmar, a serological study of birds at the largest Yangon live bird market found 4.51% seroprevalence against A/H9N2.^6^ IAVs are thought to have been introduced to Myanmar through the importation of live birds from neighboring countries,^4^ but the currently sparse surveillance limits incursion tracing.

With the threat of new influenza virus incursions in Myanmar that may be detrimental to the poultry industry and present a risk of zoonotic spillover, a partnership of the Duke Global Health Institutes (United States and Myanmar), the Livestock Breeding and Veterinary Department (LBVD) at the Ministry of Agriculture, Livestock and Irrigation, and the Central Epidemiology Unit and National Health Laboratory (NHL) at the Ministry of Health in Myanmar formed in 2018 to conduct a One Health study of zoonotic IAVs and influenza D viruses (IDVs) circulating in live bird markets in Yangon, Myanmar. A 3‐week pilot study was conducted at three major live bird markets in Yangon in July and August 2019. This is the first study of its kind to present results of paired influenza virus surveillance in birds and humans in Myanmar.

## 2 METHODS

### 2.1 Study enrollment and sample collections

This study was approved by the Duke University human subjects and animal ethics committee as well as the Institutional Review Board, Department of Medical Research, Ministry of Health, Myanmar. A 3‐week pilot data collection effort was conducted at the three largest live bird markets (Mingalar Taung Nyunt Poultry Market, Hlaing Tharyar‐1 Market, and Kyimyindine Kannar Poultry Market) in Yangon from July 15 to August 7, 2019. The live bird markets consisted of a mix of roofed and non‐roofed open‐air stalls where individual workers sold live poultry (chickens and/or ducks) that were housed in a mix of cages or pens. Birds were not butchered onsite, and other farm animals or animal products were not sold at these markets. We enrolled live bird market workers who had occupational exposure to poultry, were healthy, and were at least 18 years of age. Live bird market workers selected at random from stalls in different areas spanning each market who had not already participated in this study were asked to participate. Field teams briefed eligible participants in their native language and asked them to sign an informed consent document, permit a nasopharyngeal (NP) swab collection, and complete an enrollment questionnaire. Participants were compensated for their participation (in Myanmar, kyat 4000 or equivalent to a range of 3–4 USD). The questionnaires included questions about demographics, exposure to live animals, risk behavior for handling poultry, and the practice of using personal protective measures.

Bioaerosol sampling was conducted using the National Institute of Occupational Safety and Health's (NIOSH, Morgantown, West Virginia, USA) model BC 251 two‐stage bioaerosol sampler calibrated at a rate of 3.5 L/min. At the bird markets, three NIOSH samplers were set up in poultry‐dense sections and run for a 4‐h period during each visit. Birdcage swabs (environmental samples) were collected from poultry‐dense bottom cages, near the position of the air samplers, and placed in viral transport media. Deep oropharyngeal (OP) swabs were collected from birds, prioritizing birds that were recently sick or died, and placed in viral transport media. Bioaerosol samples were immediately processed as previously reported^7^ upon returning to the lab. Processed bioaerosol samples and swab samples were stored at −80°C prior to RNA extraction.

### 2.2 Laboratory methods

Human NP swab samples were transported on wet ice to the Virology Section Lab of NHL, and bird OP, cage, and bioaerosol samples were transported on wet ice to the Yangon Veterinary Diagnostic laboratory at LBVD where they were preserved at −80°C until ready for RNA extraction and molecular study. NHL is one of the WHO‐recognized National Influenza Centers and a member of the Global Influenza Surveillance Network. RNA extraction was performed using the QIAamp Viral RNA Mini Kit (Qiagen) per the manufacturer's instructions. RNA extracts were screened for conserved matrix genes from IAV and IDV using previously published rRT‐PCR assays.^8^, ^9^ Human NP specimens with molecular evidence of IAV were hemagglutinin‐subtyped for H1pdm and H3 using rRT‐PCR (primer and probe sequences available in Table S1). Following this, all IAV‐positive specimens were further studied at St. Jude Children's Research Hospital by culture in embryonated eggs for virus isolation. Extracted RNA from positive samples was subjected to cDNA synthesis using the Superscript IV system (Life Technologies). All eight gene segments were amplified using Phusion high‐fidelity DNA polymerase and universal conserved Uni12/13 primers for IAVs. Amplicons were purified using the GFX PCR DNA and Gel Band Purification Kit (Cytiva, UK). DNA libraries were prepared using the Nextera XT DNA Library Prep Kit (Illumina) and subsequently sequenced using an Illumina MiSeq personal genome system. The full genome of A/H9N2 viruses was assembled using CLC Genomics Workbench, version 20 (CLC Bio, Qiagen, Hilden, Germany). A/H9N2 isolates were analyzed by a hemagglutination inhibition (HI) assay against reference antisera raised against A/chicken/Hong Kong/G9/97 (H9N2), A/Hong Kong/308/2014 (H9N2), and A/Anhui‐Lujiang/39/2018 (H9N2) viruses. HI assays were performed per standard protocol with 0.5% turkey red blood cells.^10^

### 2.3 Sequence analyses

Publicly available sequences of the A/H9N2 viruses from Myanmar and reference A/H9N2 strains were downloaded from the Global Initiative on Sharing All Influenza Data (GISAID; accession numbers are available in Table S1).^11^ BLASTN homology analysis of each segment was performed on the GISAID website, and globally related sequences were downloaded. The evolutionary history of the sequences was inferred by using the Maximum Likelihood method and Kimura 2‐parameter model,^12^ which were conducted in MEGA X.^13^ Pairwise sequence analyses were performed with Jalview^14^; reference sequences included A/H9N2 sequences collected in Myanmar in 2015, HI anti‐serum strains and other related sequences from the region.

## 3 RESULTS

Our study team collected 27 bioaerosol (collected with two particle sizes, ≤4 μm and >4 μm, from each sampler), 90 bird cage, 90 bird OP, and 90 human NP samples from three major live bird markets in Yangon, Myanmar, across three weekly visits in 2019. IAV was detected in 40 of the 297 total samples collected (13.5%), and no IDV was detected. Among the IAV‐positive samples were eight bioaerosol (30.0%; four samples were positive in both particle sizes), 16 bird cage (17.8%), 15 bird OP (16.7%), and one human NP (1.1%; note, three suspected positives were detected with CT 39‐45) samples. By rRT‐PCR, one human NP swab was found to be A/H1pdm. Sequencing revealed seven IAV detections were subtype A/H9N2; efforts to type the other samples were inconclusive. All of the IAV‐positive samples were inoculated into eggs, and three were successfully isolated. These three were all subtype A/H9N2 viruses (two bird swab and one bird cage swab samples) and were subsequently fully sequenced (Genbank accession numbers pending, Table S1).

**FIGURE 1:**
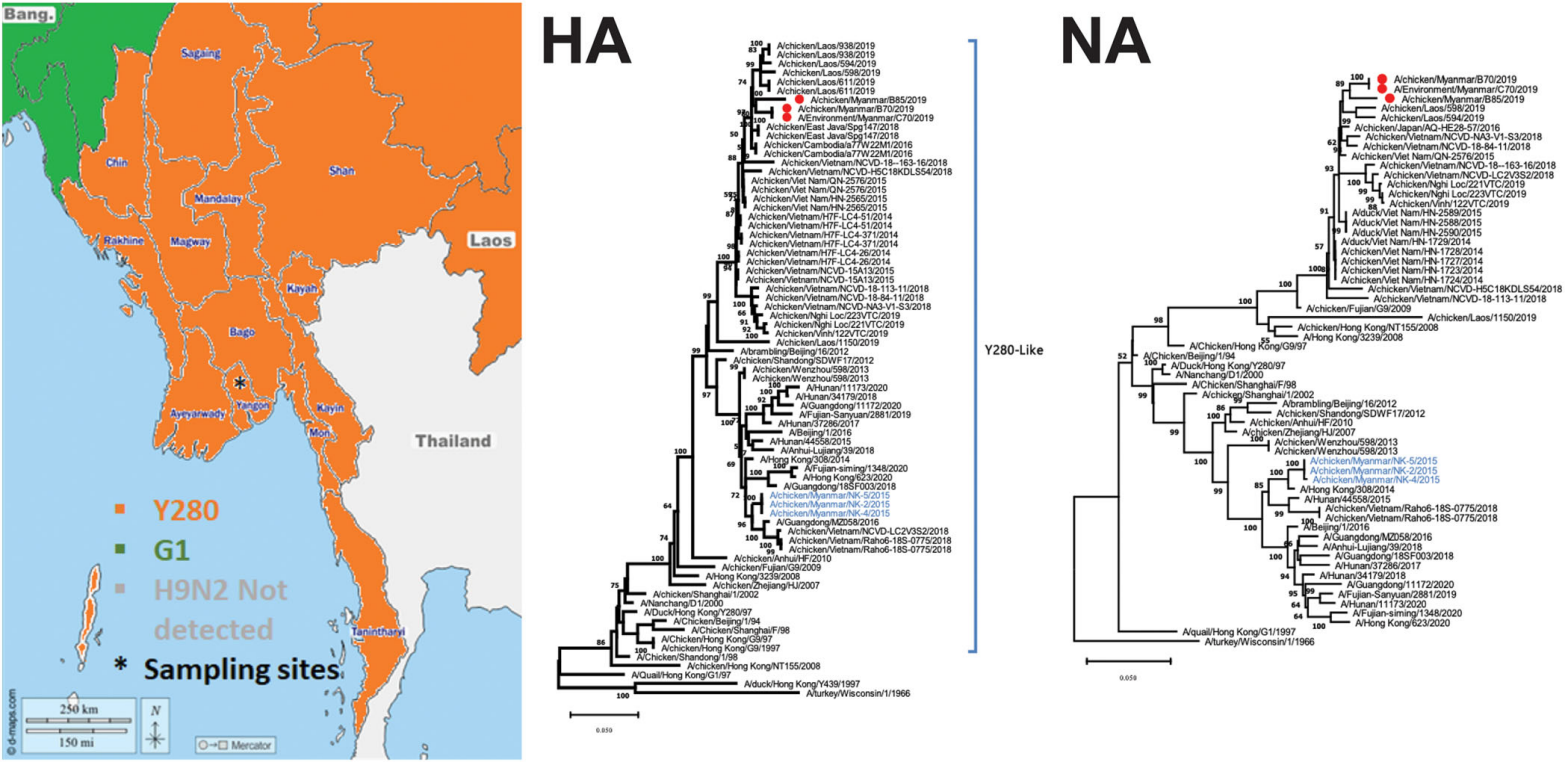
Phylogeographic map of Southeast Asia and maximum likelihood phylogenetic analysis of hemagglutinin (HA) and neuraminidase (NA) segments of isolated A/H9N2 viruses. Sampling sites are indicated by asterisks. Adjacent countries are color‐coded by reported A/H9N2 lineage detections. A/H9N2 viruses sequenced in this study are labeled with red circles. Viruses previously identified in Myanmar are colored blue.

Phylogenetic and pairwise analyses of PB2, PB1, PA, HA, NP, NA, M, and NS revealed that the viruses isolated from the Yangon bird markets in 2019 did not cluster with A/H9N2 viruses previously isolated from birds in Myanmar in 2015. The HA and NA of the Yangon bird market viruses clustered most closely with G9/Y280‐lineage A/H9N2 viruses from Southeast Asia, including Laos PDR and Vietnam (Figure 1 and Table 1). The remaining gene segments clustered most closely with viruses detected in China, Laos PDR, and Vietnam (Figure S1 and Table 1). The HAs of the characterized A/H9N2 viruses had a RSSR*GLF cleavage motif between HA1/HA2. The HA receptor binding sites contained L226/234 (H3/H9 numbering). Analyses of genetic markers associated with the virulence of A/H9N2 viruses indicated that the Myanmar A/H9N2 viruses had I504V^15^ and A588V^16^, ^17^ in PB2, I127V,^18^ N383D,^19^ and I550L^15^ in PA, P64S and L69P in M2,^18^ and A42S,^20^ F103L,^21^ M106I,^21^ V149A,^22^ and D/G189N in NS1^23^ (Table 2).

**TABLE 1:** Pairwise comparison of A/Chicken/Myanmar/B70/2019 to other H9N2 sequences collected in Myanmar and reference strains.

Strain	Nucleotide identity % (amino acid identity %)										
	PB2	PB1	PA	HA	NP	NA	M	M1	M2	NS	NS1
A/Environment/Myanmar/C70/2019	100 (100)	100 (100)	100 (100)	100 (100)	100 (100)	100 (100)	100	(100)	(100)	100	(100)
A/Chicken/Myanmar/B85/2019	96.4 (98.7)	96.7 (98.3)	97.0 (98.2)	96.8 (98.0)	97.6 (99.2)	97.4 (97.2)	98.6	(100)	(97.9)	98.3	(98.2)
A/Chicken/Myanmar/NK‐2/2015	95.9 (98.8)	94.1 (98.3)	95.8 (98.7)	93.6 (95.4)	96.0 (99.0)	83.5 (85.9)	98.0	(100)	(97.8)	94.5	(94.9)
A/Chicken/Myanmar/NK‐4/2015	95.9 (98.8)	94.1 (98.3)	95.9 (98.7)	93.6 (95.4)	96.0 (99.2)	83.5 (85.9)	98.0	(100)	(97.8)	94.5	(94.9)
A/Chicken/Myanmar/NK‐5/2015	95.7 (98.8)	94.1 (98.3)	95.7 (98.6)	93.6 (95.4)	96.0 (99.0)	83.5 (85.9)	98.0	(100)	(97.8)	94.5	(95.2)
A/Chicken/Hong Kong/G9/1997	87.5 (95.6)	88.2 (95.6)	87.4 (94.6)	90.1 (93.0)	88.0 (95.4)	89.5 (91.7)	92.2	(94.8)	(90.7)	92.7	(90.3)
A/Hong Kong/308/2014	93.9 (98.3)	94.7 (98.6)	95.5 (98.0)	93.8 (95.5)	93.2 (98.4)	83.9 (86.4)	98.1	(100)	(96.9)	97.8	(95.8)
A/Anhui‐Lujiang/39/2018	95.6 (98.4)	94.2 (98.4)	96.4 (98.5)	93.0 (95.5)	95.9 (98.8)	83.4 (85.1)	98.5	(100)	(93.8)	96.3	(94.9)
A/Chicken/China/E1562/2014	96.4 (98.8)	97.3 (99.3)	97.0 (98.9)	93.7 (95.4)	97.9 (99.0)	84.4 (86.8)	98.0	(100)	(95.9)	98.1	(96.8)
A/Chicken/China/E2620/2014	97.2 (99.5)	97.7 (99.2)	97.8 (99.0)	93.7 (95.0)	97.9 (98.8)	84.2 (86.6)	98.1	(100)	(96.9)	98.2	(96.8)
A/Chicken/China/E2838/2014	97.1 (99.5)	97.6 (99.1)	97.8 (99.0)	93.5 (95.2)	97.9 (98.8)	84.2 (86.6)	98.1	(100)	(96.9)	98.6	(97.2)
A/Chicken/Laos/594/2019	94.7 (97.5)	93.1 (98.3)	95.1 (97.9)	97.5 (97.9)	93.1 (98.8)	96.6 (96.2)	96.2	(99.2)	(95.9)	96.3	(94.9)
A/Chicken/Laos/598/2019	94.8 (97.8)	93.3 (98.4)	94.7 (97.6)	97.1 (97.9)	92.9 (98.6)	96.7 (96.8)	96.3	(99.2)	(94.8)	96.1	(94.9)
A/Chicken/Laos/611/2019	95.0 (98.0)	93.1 (97.9)	95.1 (97.9)	97.6 (98.2)	92.8 (98.6)	97.0 (97.4)	96.4	(98.8)	(94.8)	96.5	(94.9)
A/Chicken/Vietnam/H7F‐LC4–371/2014	96.3 (98.8)	93.9 (98.4)	96.0 (98.5)	97.5 (97.9)	93.9 (99.0)	98.2 (98.1)	97.4	(99.2)	(95.9)	94.6	(93.1)
A/Yuhuan/YH15/2016	95.7 (98.7)	94.9 (98.0)	96.7 (98.7)	94.4 (95.7)	93.5 (98.0)	84.1 (86.1)	99.7	(100)	(99.0)	98.1	(96.8)

*Note*: The highest identities for each segment are highlighted.

**TABLE 2:** Genetic analysis of amino acids associated with enhanced virulence of IAVs in PB2, PB1, PB1‐F2, PA, HA, NP, M2, NS1, and NS2 proteins of the H9N2 viruses characterized during the current study.

Protein	Site	Avirulent	Virulent	Tested subtypes	H9N2 Myanmar	References
PB2	147	M	L	H9N2	I	^33^
	250	V	G	H9N2	V	^33^
	292	I	V	H7N9	I	^34^
	404	F	L	H9N2	F	^35^
	504	I	V	H1N1	V	^15^
	588	A	V	H5N1 H7N9	V	^16^, ^17^
	591	Q	K	H7N9	Q	^36^
	627	E	K	H9N2	E	^33^, ^37^
	701	D	N	H1N1, H5N1	D	^38^, ^39^
PB1	317	M/V	I	(H5N1, H9N2, H7N2, H7N7), H5N1	M	^40^, ^41^
	622	D	G	H5N1	G	^42^
PA	127	I	V	H5N1	V	^18^
	224	S	P	H5N1	S	^19^
	383	N	D	H5N1	D	^19^
	550	I	L	H1N1	L	^15^
HA	Cleavage site	Monobasic	Multibasic	H5N1	RSSR*GLF	^43^
NP	286	A	V	H7N9	A	^44^
	437	T	M	H7N9	T	
M2	64	P	S/A/F	H5N1	S	^18^
	69	L	P	H5N1	P	^18^
NS1	42	A/P	S	H5N1	S	^20^
	92	D	E	H5N1	D	^41^
	103	F	L	H3N2	L	^21^
	106	M	I	H3N2	I	^21^
	149	V	A	H5N1	A	^22^
	189	D/G	N	H5N1	N	^23^
NS2	31	M	I	H5N1	M	^23^
	56	H/L	Y	H5N1	H	^23^

The isolated viruses were antigenically similar to each other, and hemagglutination was inhibited by ferret antiserum generated against the World Health Organization's candidate vaccine virus (WHO CVV) generated from A/chicken/Hong Kong/G9/97 (Table 3). Hemagglutination inhibition by antiserum raised against other G9/Y280 WHO CVVs, A/Anhui‐Lujiang/39/2018, and A/Hong Kong/308/2014 was weaker.

**TABLE 3:** Hemagglutination inhibition assay of A/H9N2 viruses isolated from Myanmar in 2019.

	A/chicken/Hong Kong/G9/1997	A/Hong Kong/308/2014	A/Anhui‐Lujiang/39/2018	Passage history
Reference antigens				
A/Chicken/Hong Kong/G9/1997	160	<	<	E8/E1/E1
A/Hong Kong/308/2014	<	1280	640	VEROCEKE1/E1
A/Anhui‐Lujiang/39/2018	<	<	2560	E2/E1/E1
Test antigens				
A/Chicken/Myanmar/B70/2019	320	<	160	E1
A/Environment/Myanmar/C70/2019	160	<	160	E1
A/Chicken/Myanmar/B85/2019	320	<	160	E1

*Note*: < denotes below limit of detection.

Of the 90 poultry workers included in this study, all were male, 84.4% were aged 18–40 years, 55.6% had middle‐high school education, and the mean duration worked at the poultry market was 6.7 years (Table 4). Two poultry workers (2.2%) were vaccinated against the human influenza virus in the last 2 years. The most common close contact with poultry reported in the last 30 days was with chickens (97.8%), followed by ducks (46.7%) and geese (2.2%) (Table 5). Family members of the poultry workers also had close contact with chickens in the last 30 days (41.1%). Self‐reported use of prevention measures showed that the most commonly used measures were handwashing with soap (93.3%) and wearing protective footwear (43.3%) (Table 6). Mask wearing was less commonly reported (40%), and wearing eye protection or dedicated clothing was rarely reported (6.6% and 4.4%, respectively). Due to the small sample sizes with few outcomes, no statistical risk factor analyses are reported.

**TABLE 4:** Socio‐demographic characteristics of poultry workers from three live bird markets in Yangon, Myanmar.

Demographic characteristics	Total *n* (*N* = 90) (%)
Age	
18–40 years	76 (84.4)
>40 years	14 (15.6)
Sex	
Female	0 (0.0)
Male	90 (100.0)
Education completed	
None	3 (3.3)
Primary school/no formal education	32 (35.6)
Middle‐high school education	50 (55.6)
University/graduate/post‐graduate education	5 (5.6)
Average monthly expenditure	
<80 000 MMK per month	4 (4.4)
80 000 MMK–120 000 MMK per month	12 (13.3)
120 001 MMK–160 000 MMK per month	26 (28.9)
>160 000 MMK per month	41 (45.6)
Did not answer	7 (7.8)
Number of cohabitants in the household, mean (SD)	3.6 (2.1)
Total years worked at the poultry market, mean (SD)	6.7 (7.8)
Received vaccination for human influenza in the past 2 years	
Yes	2 (2.2)
No	87 (96.7)
Unknown	1 (1.1)

Abbreviations: MMK, Myanmar Kyat; SD, standard deviation.

**TABLE 5:** Exposure (contact within 1 m) to live animals among poultry workers from three live bird markets in Yangon, Myanmar.

Live animals	Total (*N* = 90) (%)		
	Exposure among poultry workers in the last 30 days	Exposure among poultry workers in the last 12 months	Exposure among household members in the last 30 days
Pigs	2 (2.2)	3 (3.3)	7 (7.8)
Chickens	88 (97.8)	88 (97.8)	37 (41.1)
Ducks	42 (46.7)	47 (52.2)	10 (11.1)
Geese	2 (2.2)	2 (2.2)	1 (1.1)
Parrot	1 (1.1)	1 (1.1)	0 (0)
Dog	5 (5.6)	6 (6.7)	3 (3.3)
Cow	1 (1.1)	2 (2.2)	3 (3.3)

**TABLE 6:** Infectious disease prevention behaviors among poultry workers from three live bird markets in Yangon, Myanmar.

Behaviors	Total *n* (*N* = 90) (%)
Handwashing with soap	
Never/rarely	5 (5.6)
Sometimes/mostly/always	84 (93.3)
Eye protection used	
Goggles	2 (2.2)
Glasses	4 (4.4)
Mask used	
Dust mask	1 (1.1)
Surgical mask	36 (40.0)
Cloth mask	1 (1.1)
Protective clothing used	
Apron	6 (6.7)
Type of footwear often used	
Washable boots	39 (43.3)
Sneakers	1 (1.1)
Sandals	47 (52.2)
Gloves used	
Disposable latex or vinyl	18 (20.0)
Cloth	4 (4.4)
Leather	14 (15.6)

## 4 CONCLUSIONS

In this pilot study, we employed a One Health approach, which was unique for our collaborators in Myanmar. Public health and veterinary health professionals worked together to collect data and specimens and to perform laboratory assays. Although IDV has been detected at poultry farms in other parts of Southeast Asia,^24^ this study did not find evidence of IDV at markets in Yangon. IAV was detected in all sample types collected in this study, including bioaerosol, bird cage, bird OP, and human NP samples. IAV was found in aerosols of ≤4 μm and >4 μm but did not produce viable virus isolates in egg culturing. A/H9N2 was detected in 2.4% of the samples collected, specifically from the chicken OP and birdcage samples. A/H9N2 viruses were previously detected in Myanmar in 2015, as a speculated incursion from China.^5^ All of the IAV segment sequences identified in this study were found to be of the G9/Y280 genetic lineage, which has predominated in Southeast Asia and China over recent years.^25^ However, the A/H9N2 viruses isolated in this study do not share a recent common ancestor with the A/H9N2 viruses previously circulating in Myanmar. This is echoed by the pairwise analysis, which shows greater sequence identity between the sequences detected in this study and sequences detected in China, Vietnam, and Laos PDR compared with the sequences previously circulating in Myanmar. These findings may indicate the isolates reported here represent a separate introduction of A/H9N2 into Myanmar, which could be attributed to the importation of live birds from neighboring countries or transmission from wild bird reservoirs.

While A/H9N2 viruses have not been associated with the same number of severe zoonotic infections as A/H5NX or A/H7N9 viruses, there are a number of reasons why they are a subtype of concern. The majority of cases of A/H9N2 viral infections in humans are linked to the Y280 lineage.^26^ Moreover, the internal gene cassette of Y280 lineage viruses is thought to have been contributed to zoonotic H5, H7, and H10 influenza viruses.^27^, ^28^, ^29^ Finally, like other members of the Y280 clade, the three sequences reported in this study contain molecular markers associated with enhanced potential for mammalian spillover. The HA sequences contain a L226/234 (H3/H9 numbering) amino acid change that has been shown to confer increased affinity to α2,6‐linked sialic acid‐terminated receptors,^30^ which support binding to human respiratory epithelial cells.^31^ The sequences detected in this study also show changes in the polymerase subunit PB2 at A588V, which has been shown to enhance polymerase activity in mammalian cells.^17^

The study has a number of limitations. As a pilot study with very limited funding, data collection and analyses were necessarily limited in scope. Consequently, broad conclusions and meaningful risk factor analyses were not possible. For instance, as specimens were collected from only several bird markets over a 3‐week period, it is impossible to suggest that the findings might infer the prevalence of IAVs throughout Myanmar or for even one season in the markets studied. The prevalence estimates produced from this study may also have been influenced by the preferential selection of sick or dead birds during sample collection. While other studies have had success isolating viruses from the same model of bioaerosol samplers used in this study,^32^ the bioaerosol samples collected in this study did not yield active viruses, suggesting either a low titer or a compromised cold chain.

However, this study has value in demonstrating that human and veterinary health professionals in Myanmar can successfully work together and generate data that may prove important for both sectors. The authors were also pleased that, despite the challenges, three of the IAV‐positive samples collected from poultry yielded viruses that were characterized through sequence studies. Study data suggest that A/H9N2 viruses are likely to be prevalent in live bird markets in Myanmar. It also seems logical from these limited data that encouraging live bird market workers to use personal protective equipment might reduce their risk of suffering spillover infections with IAVs. We posit that Myanmar public health and veterinary health officials would be wise to continue to monitor live bird markets for novel influenza viruses, particularly reassortment IAVs, and zoonotic virus transmission to humans. Enhanced efforts to track and control A/H9N2 and influenza viruses generally in this region seem warranted.

## AUTHOR CONTRIBUTIONS

Funding for this study was acquired by Gregory C. Gray, Laura K. Borkenhagen, and Poe Poe Aung. This project was coordinated by Poe Poe Aung, Laura K. Borkenhagen, and Thet Su Mon. Sample collection was overseen by Thura Htay. Laboratory methodologies were optimized by Emily S. Bailey. Laboratory analyses were performed by Win Myint, Ommar Swe Tin, and Ahmed M. Kandeil. Zaw Win Thein was responsible for data management. Phylogenetic analyses were performed by Ahmed M. Kandeil. Laboratory and phylogenetic analyses at St. Jude were overseen by Richard J. Webby. The original draft of this work was generated by Timothy G. Wanninger and Laura K. Borkenhagen. The final draft was reviewed, edited, and approved by all authors.

## CONFLICT OF INTEREST

The authors report no conflicts of interest.

### PEER REVIEW

The peer review history for this article is available at https://publons.com/publon/10.1111/irv.13111.

## Supporting information


**Table S1.** Primers and probes used for influenza A virus hemagglutinin subtyping.
**Table S2.** Record of influenza A virus‐positive specimens collected from three poultry markets in Yangon, Myanmar, in 2019.
**Table S3.** List of GISAID sequences used for phylogenetic analyses.

**Figure S1.** Maximum Likelihood analysis of PB2, PB1, PA, NP, M, and NS segments of isolated H9N2 viruses. H9N2 viruses sequenced specifically for this study are labeled with red circles. Viruses previously identified in Myanmar are colored blue.

## Data Availability

The findings of this study are based on metadata associated with sequences available on GISAID. Accession numbers are available in Table S1.
