# Supplementary figures and images for: A cross‐sectional study of avian influenza A virus in Myanmar live bird markets: Detection of a newly introduced H9N2?
Source: Influenza Other Respir Viruses. 2023 Feb 22;17(2):e13111. doi: 10.1111/irv.13111 (PMC9970615; doi:10.1111/irv.13111)

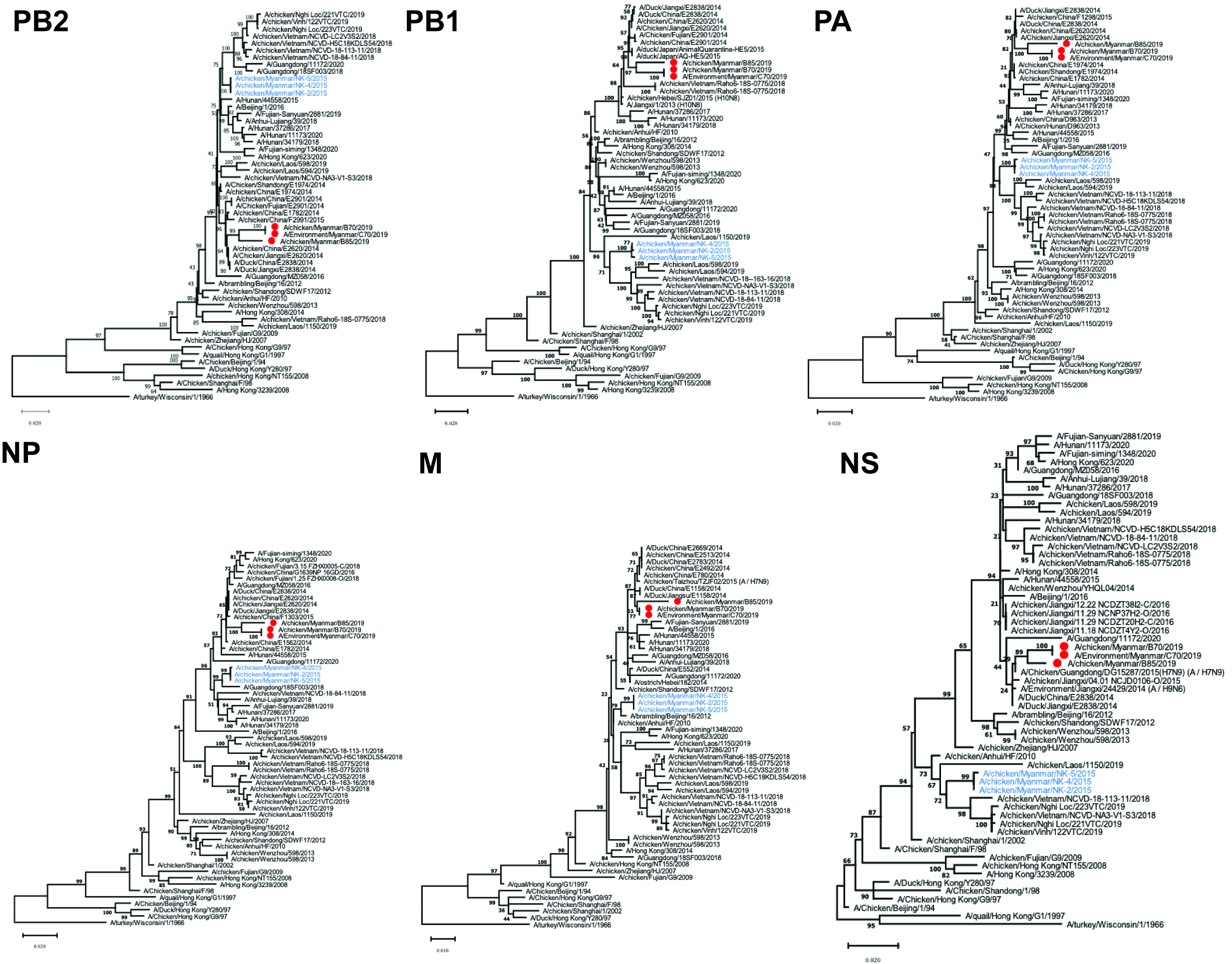

Supplement: Supplementary file 2 — Figure S1. Maximum Likelihood analysis of PB2, PB1, PA, NP, M, and NS segments of isolated H9N2 viruses. H9N2 viruses sequenced specifically for this study are labeled with red circles. Viruses previously identified in Myanmar are colored blue. [file IRV-17-e13111-s001.tif]
